# Investigating the relationship between inflammatory cytokines and adolescent depression: a comparative analysis

**DOI:** 10.3389/fpsyt.2025.1524015

**Published:** 2025-02-06

**Authors:** Mengqi Liu, Jie Tang, Gaoyang Xu, Xu Chen, Kun Fang, Fan He, Yi Zheng

**Affiliations:** ^1^ The National Clinical Research Center for Mental Disorders & Beijing Key Laboratory of Mental Disorders, Beijing Anding Hospital, Capital Medical University, Beijing, China; ^2^ Advanced Innovation Center for Human Brain Protection, Capital Medical University, Beijing, China; ^3^ School of Basic Medical Sciences, Capital Medical University, Beijing, China

**Keywords:** adolescents, inflammation, depression, TNF-α, IL-4

## Abstract

**Background:**

Major depressive disorder (MDD) in adolescents poses a significant threat to physical health. Previous studies have indicated that adolescents with MDD exhibit immune activation.

**Objective:**

This study aimed to compare cytokine levels in adolescents with MDD to healthy controls and assess their diagnostic value.

**Methods:**

This cross-sectional study included 58 adolescent patients with depression from Beijing Anding Hospital (outpatients and inpatients) and 40 healthy adolescents recruited from the community. Multiplex cytokine analysis (Luminex xMAP) was used to measure serum levels of several cytokines. Data analysis was performed using SPSS (version 26.0) to compare cytokine levels between adolescents with MDD and healthy controls and assess gender differences. Correlations between cytokine levels and demographic data, clinical features and depressive symptoms were also analyzed. The receiver operating characteristic (ROC) curves were used to evaluate the diagnostic value of cytokines.

**Results:**

Serum IL-4, IFN-γ, and TNF-α levels were significantly elevated in MDD group compared with healthy controls (*p* < 0.05). In MDD group, the age of first onset of depression in females was significantly younger than that in males (*p* < 0.05), and female serum TNF-α levels were significantly higher than those in males (*p* < 0.05). BMI and serum IL-4 levels were significantly positively correlated in adolescents with MDD. The area under the ROC curve for serum IL-4 and TNF-α in diagnosing adolescent depression was 0.695 (95% confidence interval [CI]: 0.580 - 0.809; *p* < 0.05), with a sensitivity of 0.793 and specificity of 0.675.

**Conclusion:**

Compared with healthy controls, adolescents with depression demonstrated significantly elevated serum cytokine levels, indicating immune activation which were higher in female. Cytokines may have promising diagnostic value in adolescent depression, but further validation with additional indicators is needed.

## 1 Introduction

Major depressive disorder (MDD) is characterized by persistent poor mood, diminished interest, and lack of energy, with high prevalence, recurrence, and disability rates. It is a leading cause of disability worldwide (1). Depression can occur at any age; however, the risk is especially high during adolescence (2). Adolescent depression can lead to decreased academic performance, impaired social functioning, increased suicide risks, substance abuse, and depression recurrence in adulthood (2).

Currently, adolescents with MDD encounter challenges such as low diagnosis rates and poor drug treatment outcomes (3, 4). Adolescent depression is often overlooked, which can be confused with irritability. Additionally, it may manifest as unexplained physical symptoms, eating disorders, anxiety, behavioral problems, truancy, academic decline, and substance abuse, further complicating diagnosis (5). As for treatment, traditional monoamine-targeted antidepressants have a slow onset (approximately 2 to 4 weeks), low clinical cure rates, significant residual symptoms, and higher risks of relapse and functional impairment (6). Understanding the underlying mechanisms of adolescent depression is crucial for developing novel diagnostic methods and improved treatment strategies to mitigate its adverse effects.

Inflammatory cytokines are a class of soluble proteins that regulate immune responses, cell proliferation, and tissue repair (7). Based on their effects, cytokines are classified as pro-inflammatory, including interleukin-1 (IL-1), tumor necrosis factor-alpha (TNF-α), IL-6, and IL-12, and anti-inflammatory, including IL-4, IL-10, and IL-13. They are further classified structurally and functionally as interleukins, interferons, tumor necrosis factor superfamily members, colony-stimulating factors, chemokines, and growth factors. Dysregulation of inflammatory cytokines is linked to several diseases, including severe infections and autoimmune disorders. A close relationship exists between inflammatory cytokines and mental illnesses, including depression (8, 9).

Depression, a condition characterized by immune system hyperactivity, is associated with elevated levels of inflammatory cytokines (10). Compared with healthy individuals, patients with depression exhibit significantly higher serum/plasma levels of pro-inflammatory cytokines such as IL-6 and C-reactive protein (CRP) (8). Furthermore, the severity of depressive symptoms correlates with inflammatory cytokine levels in the bloodstream (11). Additionally, patients with depression exhibit a decrease in serum/plasma cytokine levels following treatment (12). Inflammatory cytokines can cross the blood-brain barrier, altering neurotransmitter metabolism and neurogenesis and thereby contributing to depressive symptoms (13). Consistent with findings in adults, a meta-analysis of 21 studies conducted by Marlena et al. indicated a correlation between adolescent depression and elevated levels of CRP and IL-6 (14). However, research on the relationship between adolescent depression and inflammation remains limited. During puberty, lymphoid tissue shrinks, and sex hormone release increases, potentially affecting inflammatory cytokine levels, thus necessitating a focused investigation into the relationship between adolescent depression and inflammation. Furthermore, existing studies exhibit considerable heterogeneity, partly due to various cytokine-influencing factors, such as medication status, tobacco and alcohol use, and comorbid neurodevelopmental disorders, including pervasive developmental disorders and attention-deficit hyperactivity disorder. Future studies must seek to adjust for these confounding variables as much as possible.

Based on previous research on inflammatory levels in adolescent depression, we hypothesized that adolescents with depression demonstrate immune activation and abnormal serum inflammatory cytokine levels compared with healthy controls. This study aimed to compare serum inflammatory cytokine levels between adolescents with MDD and healthy controls, analyze gender differences, explore the correlation between cytokine levels and other variables, and assess the diagnostic value of inflammatory cytokine levels for adolescent depression. This research could provide insights into the pathogenesis of adolescent depression, promoting the identification of effective diagnostic and treatment methods.

## 2 Materials and methods

### 2.1 Participants

**Figure 1:**
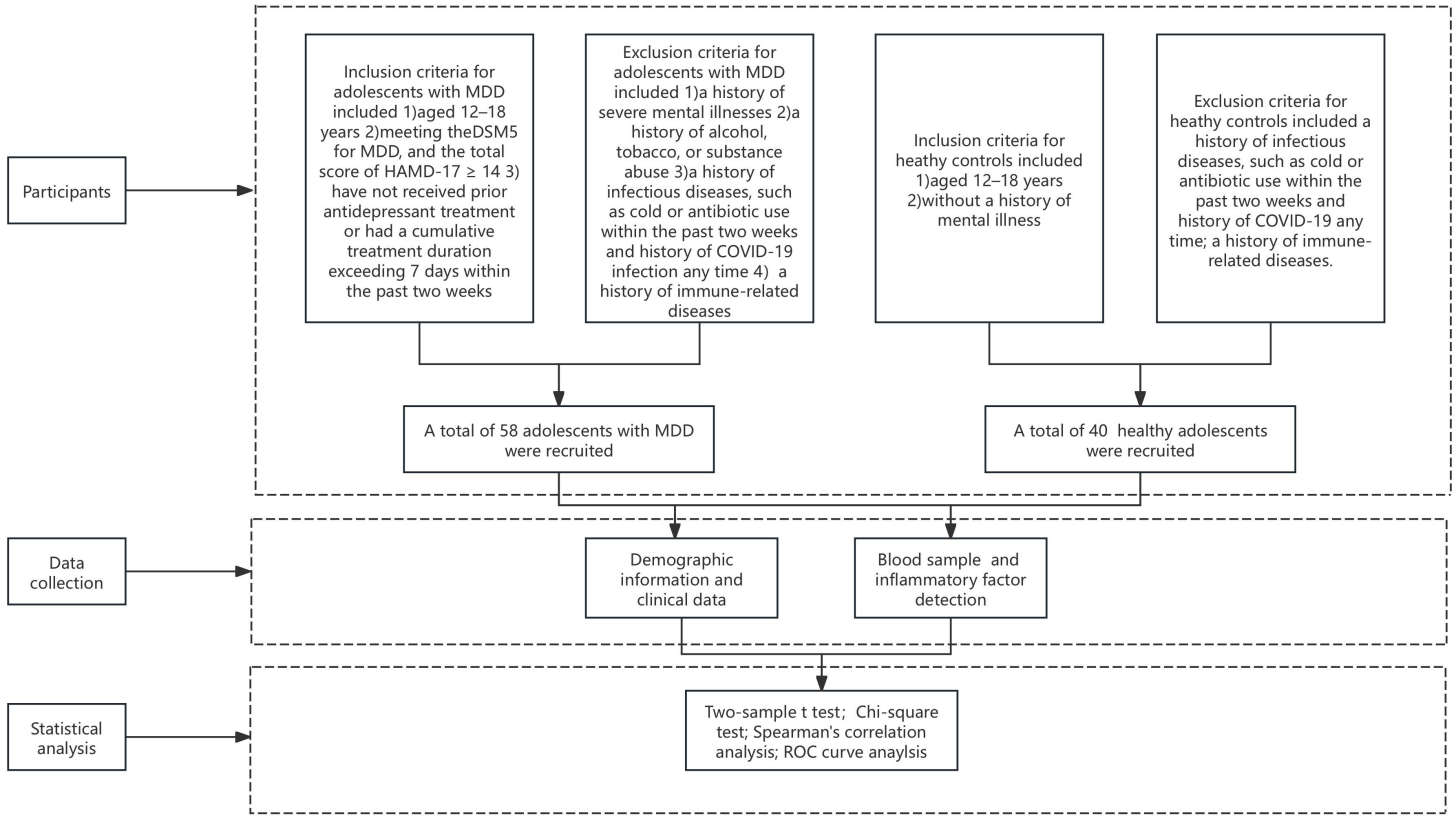
The flowchart of our study.

This study used a cross-sectional design, with the case group comprising adolescents diagnosed with MDD who sought outpatient and inpatient care at Beijing Anding Hospital, Capital Medical University, between July 2022 and February 2023. The research process was shown in **Figure 1**. The healthy control group comprised adolescents recruited from the community during the same period. Ethical approval was granted by the Ethics Committee of Beijing Anding Hospital [(2022) Research No (104).-2022136FS-2]. Inclusion criteria for adolescents with MDD included being aged 12–18 years, meeting the Diagnostic and Statistical Manual of Mental Disorders, Fifth Edition diagnostic criteria for a major depressive episode, and exhibiting a Hamilton Depression Scale (HAMD-17) score of ≥ 14. Participants must not have received prior antidepressant treatment or had a cumulative treatment duration exceeding 7 days within the past two weeks (participants include those who have received no prior antidepressant treatment at all, who have had no treatment during the last two weeks and who have had no more than seven individual days of treatment over the last fourteen days). Exclusion criteria included a history of severe mental illnesses, such as schizophrenia, schizoaffective disorder, or intellectual disability; a history of alcohol, tobacco, or substance abuse; a history of infectious diseases, such as cold or antibiotic use within the past two weeks and history of COVID-19 infection any time and a history of immune-related diseases.

Inclusion criteria for healthy controls included being aged 12–18 years, and without a history of mental illness. Exclusion criteria included a history of infectious diseases, such as cold or antibiotic use within the past two weeks and history of COVID-19 any time and a history of immune-related diseases.

Furthermore, all adolescents and their guardians were required to cooperate and provide informed consent.

### 2.2 Demographic information and clinical data collection

A self-designed demographic information questionnaire was used to collect general demographic data from patients and healthy control groups, including name, gender, age, height, weight, family structure, age at first depressive episode, history of previous psychiatric diagnoses, psychiatric medication use, family history, history of physical illnesses and age of first onset.

### 2.3 Depressive symptoms

The HAMD-17 was conducted independently by one psychiatrist or rater. Before the study commenced, all raters were briefed on the study protocol and trained on scale consistency. The HAMD-17, developed by Hamilton, is the most commonly used scale for assessing depressive states in clinical practice (15). It evaluates the severity of depressive symptoms and treatment response over the past week. The HAMD-17 includes 17 items categorized into five factors: anxiety and somatization, cognitive disturbance, retardation, weight, and sleep disturbances. Most items are rated on a 0 to 4 scale, while a few are scored from 0 to 2. The total score is calculated by summing the individual item scores. The Chinese version of the HAMD-17 has a Cronbach’s α coefficient of 0.714 (16), accurately reflecting the severity of depression in patients.

### 2.4 Blood sample collection

On the day of assessment, all participants provided fasting peripheral blood samples (5 mL) in serum collection tubes (yellow cap) between 6:00 and 10:00 AM. To separate serum, after blood collection, samples were gently mixed 8-10 times and sat upright for a minimum of 30 minutes to a maximum of one hour to allow the blood to clot at room temperature (R/T). Centrifugation conditions were 1,500-2,000 x g for 10 minutes. The serum was aliquoted and stored at −80°C in the biorepository of Beijing Anding Hospital, Capital Medical University, for future analysis.

### 2.5 Serum inflammatory factor detection

Serum inflammatory factors were evaluated using the ProcartaPlex™ immunoassay technology with a human serum inflammatory factor kit (catalog number: EPX200-12185-901; manufacturer: Thermo Fisher Scientific) on a Luminex 200 platform. This technology quantifies serum levels of intercellular adhesion molecule-1 (ICAM-1), IL-1α, IL-1β, IL-4, IL-6, IL-8, IL-10, IL-12p70, IL-13, IL-17A, interferon-alpha (IFN-α), interferon-gamma (IFN-γ), interferon-inducible protein-10 (IP-10), monocyte chemotactic protein-1 (MCP-1), macrophage inflammatory protein-1α (MIP-1α), MIP-1β, E-selectin, P-selectin, and TNF-α and Granulocyte–macrophage colony-stimulating factor (GM-CSF). While IL-1α, IL-1β, IL-6, IL-10, IL-13, IL-17A, IFN-α and GM-CSF did not meet the detection standards. ProcartaPlex™ assays use a sandwich enzyme-linked immunosorbent assay approach, with two highly specific antibodies binding to different epitopes of the target protein, allowing for simultaneous quantification of all analytes. Multiplex assays require a minimum of 25 µL of serum, and results can be obtained within 4 h. Standard samples (in duplicate) were used to generate calibration curves for each analyte, and quality control samples were used to monitor assay performance, ensuring inter-assay variation of less than 15%; otherwise, the experiment was considered unsuccessful and repeated upon identifying the issue.

### 2.6 Statistical methods

Data analysis was performed using Statistical Package for the Social Sciences software (version 26.0). A one-sample Kolmogorov–Smirnov test was used to assess the normality. Because the cytokine levels did not follow normal distribution, a logarithmic transformation was applied. Independent samples t-tests were used to compare continuous data between two groups. Spearman’s correlation analysis was used to investigate the relationships between demographic, clinical characteristics and total HAMD-17 score and inflammatory factor levels. Receiver operating characteristic (ROC) curves were used to assess the diagnostic value of inflammatory factors for adolescents with MDD. A *p* < 0.05 (two-tailed) was considered statistically significant.

## 3 Results

### 3.1 Comparison of demographic data and serum inflammatory factors between adolescents with depression and healthy controls

This study included 58 adolescents with MDD and 40 healthy controls. The mean age of the adolescent depression group (N = 58) was 14.41 ± 1.40 years, comprising 14 males (24.13%) and 44 females (75.86%), with an average body mass index (BMI) of 21.58 ± 4.12 kg/m². The healthy control group (N = 40) had a mean age of 14.43 ± 1.64 years, with 11 males (27.50%) and 29 females (72.50%). No significant differences were observed between the two groups in age, gender, or BMI.

**Figure 2:**
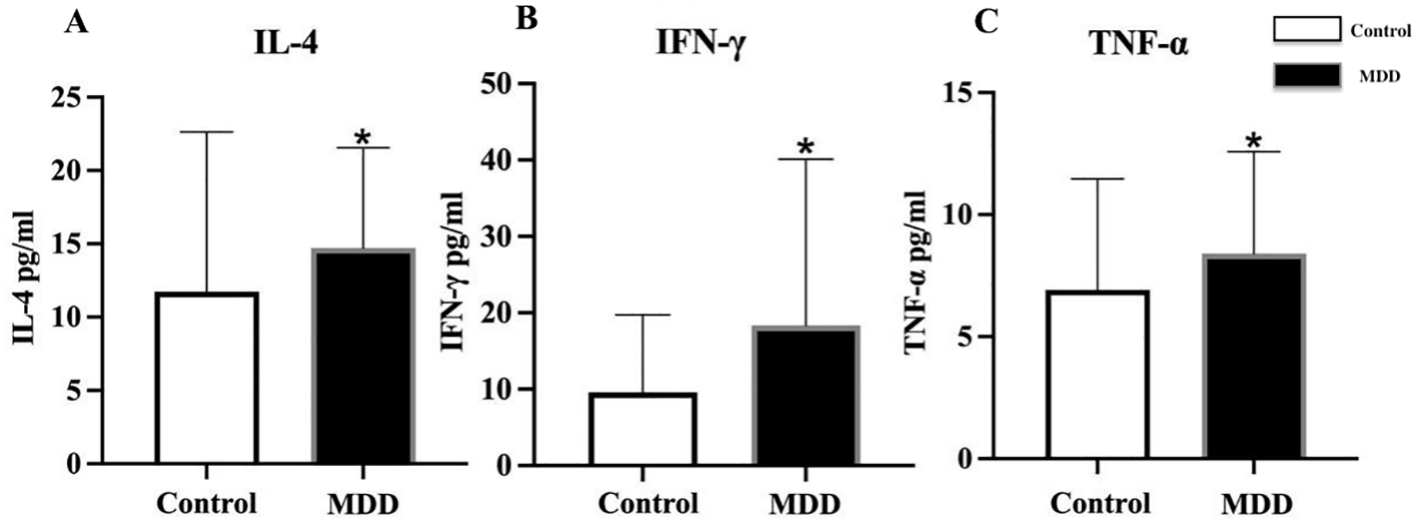
Comparison of serum inflammatory factor levels between adolescents with depression and healthy controls. MDD, major depressive disorder; Control, healthy control group; IL-4, interleukin-4; IFN-γ, interferon-γ; TNF-α, tumor necrosis factor-α. *indicates p < 0.05 compared with Control. All inflammatory factors are presented as mean ± standard deviation. Differences in serum **(A)** IL-4, **(B)** IFN-γ, and **(C)** TNF-α between healthy controls and MDD adolescents.

For serum inflammatory factors, serum levels were first transformed using logarithmic transformation to meet normality assumptions. Adolescents with depression exhibited significantly higher serum levels of IL-4, IFN-γ, and TNF-α compared with healthy controls (IL-4: 14.71 ± 6.83 *vs.* 11.73 ± 10.90 pg/mL, t = 2.859, *p* = 0.006; IFN-γ: 18.34 ± 21.75 *vs.* 9.60 ± 10.13 pg/mL, t = 2.058, *p* = 0.042; TNF-α: 8.41 ± 4.17 *vs.* 6.92 ± 4.54, t = 2.324, *p* = 0.018) (**Figures 2A–C**). No significant differences were observed in the serum levels of ICAM-1, IL-8, IL-12p70, IP-10, MCP-1, MIP-1α, MIP-1β, E-selectin, and P-selectin between the two groups (**Table 1**).

**Table 1:** Comparison of demographics, and serum inflammatory factors between adolescents with depression and healthy controls.

Variables	MDD (N=58)	Control (N=40)	*χ^2^/t*	*p*
Age (years)	14.41 ± 1.40	14.43 ± 1.64	−0.036	0.971
Sex				
Male (%)	14 (24.13%)	11 (27.50%)	0.141	0.707
Female (%)	44 (75.86%)	29 (72.50%)		
BMI (kg/m^2^)	21.58 ± 4.12	20.99 ± 3.33	0.760	0.449
ICAM-1^⋄^ (pg/mL)	111195.04 ± 80895.61	82711.961 ± 64275.86	1.511	0.134
IL-4^⋄^ (pg/mL)	14.71 ± 6.83	11.73 ± 10.90	2.859	0.006*
IL-8^⋄^ (pg/mL)	2.39 ± 2.89	2.37 ± 3.31	0.442	0.659
IL-12p70^⋄^ (pg/mL)	20.54 ± 21.07	14.47 ± 10.71	1.037	0.303
IP-10^⋄^ (pg/mL)	10.55 ± 8.16	11.62 ± 9.66	−0.521	0.604
IFN-γ^⋄^ (pg/mL)	18.34 ± 21.75	9.60 ± 10.13	2.058	0.042*
MCP-1^⋄^ (pg/mL)	60.01 ± 30.07	59.28 ± 38.70	0.830	0.409
MIP-1α^⋄^ (pg/mL)	9.04 ± 4.94	10.85 ± 12.69	1.275	0.208
MIP-1β^⋄^ (pg/mL)	83.80 ± 41.09	83.01 ± 40.69	0.201	0.841
sESelectin	15096.81 ± 5883.99	15572.12 ± 5602.75	−0.583	0.561
sPSelectin	344151.46 ± 356323.65	311340.14 ± 320462.36	0.598	0.551
TNF-α^⋄^ (pg/mL)	8.41 ± 4.17	6.92 ± 4.54	2.324	0.018*

BMI, body mass index; HAMD-17, Hamilton Depression Scale 17; ICAM, intercellular adhesion molecule; IL, interleukin; IFN, interferon; MCP, monocyte chemoattractant protein; IP-10, interferon-γ-inducible protein 10; MIP, macrophage inflammatory protein; TNF, tumor necrosis factor. Data are presented as mean ± standard deviation or number (%) ⋄ indicates that serum inflammatory factor levels were log-transformed to meet normal distribution. *p < 0.05.

### 3.2 Gender differences

In the MDD group (**Table 2**), the age of first onset in females was significantly younger than that in males (12.98 ± 1.42 *vs.* 13.93 ± 1.44 years, *t* = -2.174, *p* = 0.034). Females with MDD exhibited higher serum TNF-α levels than males (8.90 ± 4.21 *vs.* 6.88 ± 3.77 pg/mL, t = 2.072, *p* = 0.043). While no differences in gender were observed in the healthy control group (**Table 3**).

**Table 2:** Differences in demographic, clinical characteristics and depressive symptoms and serum inflammatory factors in the MDD group.

Variables	Female (N=44)	Male (N=14)	*t*	*p*
Age (years)	14.32 ± 1.39	14.71 ± 1.54	-0.903	0.370
BMI (kg/m^2^)	21.17 ± 3.70	22.90 ± 5.18	-1.159	0.262
Age of first onset (years)	12.98 ± 1.42	13.93 ± 1.44	-2.174	0.034*
HAMD-17	22.48 ± 2.91	22.79 ± 3.12	-0.339	0.736
ICAM1 (pg/mL)	117268.72 ± 82181.96	92106.33 ± 76396.08	0.321	0.75
IFN-γ (pg/mL)	19.13 ± 22.34	15.89 ± 20.38	0.877	0.384
IL-12p70 (pg/mL)	21.66 ± 22.68	17.02 ± 15.09	0.453	0.652
IL-4 (pg/mL)	14.24 ± 6.87	16.21 ± 6.78	-1.087	0.282
IL-8 (pg/mL)	2.13 ± 1.62	3.20 ± 5.20	-0.44	0.662
IP-10 (pg/mL)	10.74 ± 8.66	9.97 ± 6.60	0.323	0.748
MCP-1 (pg/mL)	61.16 ± 31.70	56.41 ± 24.97	0.084	0.933
MIP-1α (pg/mL)	9.50 ± 4.70	7.62 ± 5.55	1.79	0.092
MIP-1β (pg/mL)	84.51 ± 41.71	81.60 ± 40.50	0.271	0.787
sESelectin (pg/mL)	15354.36 ± 5857.44	14287.38 ± 6114.37	0.484	0.63
sPSelectin (pg/mL)	386681.18 ± 384314.01	210486.63 ± 206881.00	1.251	0.216
TNF-α (pg/mL)	8.90 ± 4.21	6.88 ± 3.77	2.072	0.043*

BMI, body mass index; HAMD-17, Hamilton Depression Scale 17; ICAM, intercellular adhesion molecule; IL, interleukin; IFN, interferon; MCP, monocyte chemoattractant protein; IP-10, interferon-γ-inducible protein 10; MIP, macrophage inflammatory protein; TNF, tumor necrosis factor. *p<0.05.

**Table 3:** Differences in demographic characteristics and serum inflammatory factors in the healthy control group.

Variables	Female (N=29)	Male (N=11)	*t*	*p*
Age (years)	14.34 ± 1.76	14.64 ± 1.36	-0.495	0.623
BMI (kg/m^2^)	20.82 ± 3.73	21.44 ± 1.97	-0.525	0.603
ICAM1 (pg/mL)	78751.86 ± 60509.65	93152.23 ± 75465.62	-0.265	0.792
IFN-γ (pg/mL)	9.97 ± 11.21	8.60 ± 6.83	0.449	0.656
IL-12p70 (pg/mL)	15.42 ± 11.64	11.99 ± 7.65	0.47	0.641
IL-4 (pg/mL)	10.41 ± 10.48	15.23 ± 11.72	-1.637	0.11
IL-8 (pg/mL)	2.42 ± 3.66	2.26 ± 2.26	0.198	0.844
IP-10 (pg/mL)	12.10 ± 10.55	10.38 ± 7.06	0.426	0.672
MCP-1 (pg/mL)	55.81 ± 35.34	68.43 ± 47.10	-0.753	0.456
MIP-1α (pg/mL)	10.48 ± 12.20	11.81 ± 14.49	-0.246	0.807
MIP-1β (pg/mL)	86.79 ± 41.19	73.05 ± 39.45	0.92	0.363
sESelectin (pg/mL)	15583.44 ± 5361.25	15542.30 ± 6476.29	0.197	0.845
sPSelectin (pg/mL)	304400.29 ± 292270.48	329636.09 ± 401030.32	0.28	0.781
TNF-α (pg/mL)	6.44 ± 4.64	8.20 ± 4.21	-1.576	0.123

BMI, body mass index; ICAM, intercellular adhesion molecule; IL, interleukin; IFN, interferon; MCP, monocyte chemoattractant protein; IP-10, interferon-γ-inducible protein 10; MIP, macrophage inflammatory protein; TNF, tumor necrosis factor.

### 3.3 Correlation analysis

In the MDD group (**Table 4**), BMI was significantly positively correlated with serum IL-4 levels (*r_s_* =0.285, *p*<0.05). No significant correlations were observed between serum cytokine levels and age, age at first onset, or HAMD-17 total score. In the healthy control group (**Table 5**), no significant correlations were found between serum cytokine levels and other variables.

**Table 4:** Correlation analysis of serum inflammatory factors in the MDD group.

Variables	Age	Age of first onset	BMI	HAMD17
ICAM1	0.042	-0.072	0.249	0.059
IFN-γ	-0.037	0.113	0.117	0.166
IL-12p70	0.136	0.240	0.014	0.019
IL-4	-0.110	-0.016	0.285*	-0.175
IL-8	-0.111	-0.114	0.074	0.169
IP-10	-0.157	0.008	-0.058	-0.107
MCP-1	0.028	-0.165	0.154	0.223
MIP-1α	-0.204	-0.119	-0.033	0.04
MIP-1β	-0.207	-0.191	0.106	0.111
sESelectin	-0.073	-0.025	-0.014	0.042
sPSelectin	-0.213	-0.081	0.155	0.019
TNF-α	-0.125	-0.245	0.114	0.090

BMI, body mass index; ICAM, intercellular adhesion molecule; IL, interleukin; IFN, interferon; MCP, monocyte chemoattractant protein; IP-10, interferon-γ-inducible protein 10; MIP, macrophage inflammatory protein; TNF, tumor necrosis factor. *p<0.05.

**Table 5:** Correlation analysis of serum inflammatory factors in the healthy control group.

Variables	Age	BMI
ICAM1	-0.126	-0.04
IFN-γ	-0.013	0.135
IL-12p70	-0.048	0.158
IL-4	-0.153	-0.107
IL-8	-0.138	-0.04
IP-10	-0.098	0.004
MCP-1	0.02	-0.015
MIP-1α	0.148	0.097
MIP-1β	0.045	-0.002
sESelectin	0.046	0.131
sPSelectin	-0.162	0.014
TNF-α	0.168	-0.106

BMI, body mass index; ICAM, intercellular adhesion molecule; IL, interleukin; IFN, interferon; MCP, monocyte chemoattractant protein; IP-10, interferon-γ-inducible protein 10; MIP, macrophage inflammatory protein; TNF, tumor necrosis factor.

### 3.4 Diagnostic value of serum inflammatory factors in adolescents with depression

**Figure 3:**
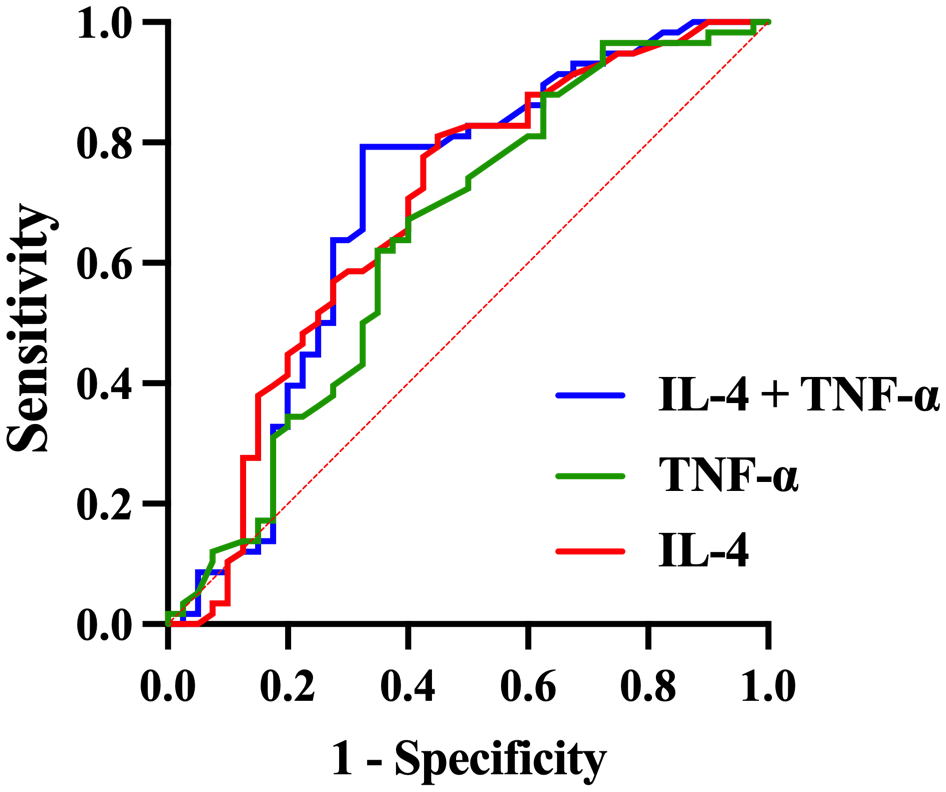
ROC curve for the diagnosis of adolescent depression patients using combined inflammatory factors.

ROC curve analysis was performed to compare the adolescent depression group and healthy control group, evaluating the diagnostic value of serum TNF-α, IL-4, and IFN-γ levels for adolescent depression. The area under the curve (AUC) of IFN-γ was 0.614 (95% confidence interval [CI]: 0.503 - 0.725, *p* = 0.056), with a sensitivity of 0.517 and a specificity of 0.750 (**Table 6**, **Figure 3**). The AUC of IL-4 was 0.686 (95%CI: 0.573 - 0.798, *p* =0.002), with a sensitivity of 0.810 and a specificity of 0.550. The AUC of TNF-α was 0.642 (95%CI: 0.527 - 0.758, *p* =0.017), with a sensitivity of 0.672 and a specificity of 0.600. The AUC of IL-4 combined TNF-α was 0.695 (95%CI: 0.580 - 0.809, *p* =0.001), with a sensitivity of 0.793 and a specificity of 0.675.

**Table 6:** ROC curve analysis for Serum inflammatory factors in diagnosing adolescent depression.

Variables	AUC	*p*	95% CI	Youden’s index	Sensitivity	Specificity
IFN-γ	0.614	0.056	0.503 - 0.725	0.267	0.517	0.750
IL-4	0.686	0.002**	0.573 - 0.798	0.360	0.810	0.550
TNF-α	0.642	0.017*	0.527 - 0.758	0.272	0.672	0.600
IL-4 + TNF-α	0.695	0.001**	0.580 - 0.809	0.468	0.793	0.675

IL, interleukin; IFN, interferon; MCP, monocyte chemoattractant protein; TNF, tumor necrosis factor. *p<0.05; **p<0.01.

## 4 Discussion

Adolescent depression is difficult to treat and is associated with high rates of disability, significantly affecting psychological and physical functioning. However, the underlying mechanisms of this condition remain unclear. Our investigation of serum inflammatory factor levels contributes to a better understanding of its immunological pathogenesis.

Adolescents with depression exhibited significantly higher serum levels of TNF-α compared with healthy controls, which is consistent with previous studies on adolescent MDD and TNF-α (17–19). This finding is also corroborated by investigations into adult MDD (20). TNF-α mediates depressive symptoms through three primary mechanisms: First, it can upregulate levels of peripheral adrenocorticotropic hormone, corticotropin-releasing hormone, and glucocorticoids, resulting in increased activity of the hypothalamic-pituitary-adrenal axis and subsequent depressive symptoms (21). Second, TNF-α activates the serotonin transporter, resulting in decreased serotonin levels, which contributes to depressive symptoms (22–24). Third, TNF-α activates indoleamine 2,3-dioxygenase (IDO), which inhibits serotonin production through the tryptophan-kynurenine pathway. The products of the kynurenine pathway, such as quinolinic acid, are neurotoxic and exacerbate depressive symptoms (25, 26). Given the role of TNF-α in depression, anti-TNF-α biologics may exert antidepressant effects (27) (28). Our findings highlight the role of TNF-α in adolescent depression, suggesting that its levels may be reduced using anti-TNF-α biologics or non-steroidal anti-inflammatory medications. This indicates that these anti-inflammatory agents could possess antidepressant properties. Future research should focus on these avenues to discover new treatment options for adolescent depression.

Adolescents with depression demonstrated higher serum IFN-γ levels, which is consistent with previous studies (17, 29). IFN-γ is associated with depression through two primary mechanisms: Similar to TNF-α, it can activate IDO, altering serotonin levels and producing neurotoxins. Additionally, IFN-γ can induce the generation of reactive oxygen species, reducing tetrahydrobiopterin levels. This reduces catecholamine synthesis and disrupts neurotransmitter signaling, contributing to depressive symptoms (30). Animal studies have demonstrated that IFN-γ can induce microglial damage to hippocampal neurogenesis, resulting in depressive-like behaviors and cognitive impairments (31). Our findings corroborate the notion that adolescent depression is characterized by immune hyperactivity, with elevated inflammatory factors playing a role in its pathogenesis. Future studies may demonstrate that inflammation plays a critical role in the diagnosis, treatment efficacy prediction, and management of adolescent depression.

IL-4, an anti-inflammatory cytokine, is a key component of the compensatory immune response system (CIRS), regulating the inflammatory response system (IRS) represented by TNF-α (32). Depression activates the IRS, which produces pro-inflammatory factors such as TNF-α and IL-6. The CIRS releases anti-inflammatory factors such as IL-4 to regulate inflammation and maintain immune balance (32). IL-4 mediates the activation of M2 macrophages, which release transforming growth factor-β, soluble IL-1 receptor antagonist, and IL-10 to reduce pro-inflammatory activity. This plays an essential role in controlling the inflammatory response. Our study aligns with the findings of Nikola et al. on adolescents (17, 33) and Pavón et al. on adults (34–36), indicating that serum IL-4 levels are elevated in adolescents with depression. This suggests that anti-inflammatory factors also play an important role in the pathogenesis of adolescent depression, where pro-inflammatory and anti-inflammatory factors are activated to help regulate immune balance.

Consistent with most previous studies, we discovered no association between inflammatory factors and the severity of depressive symptoms in adolescents (37). However, such a correlation has been observed in adult populations (38), implying that this relationship may be age-dependent. Regarding gender differences, we found that female adolescents with MDD had significantly higher serum TNF-α levels compared males. This suggests that gender may modulate the immune-inflammatory response in depression which may be related to sex hormones. Testosterone possesses anti-inflammatory properties (39) while Estrogen exhibits a U-shaped effect on inflammation (40). The significant fluctuations in estrogen levels during the female menstrual cycle lead to changes in inflammation levels which may further increase the likelihood of depression when facing negative social events (40).Gender-based analyses have shown that female patients with depression have higher levels of CRP and IL-6 compared to healthy females (41). Further research is needed to assess the impact of gender on adolescents with depression. Our study also found a negative correlation between BMI and IL-4 levels in MDD group. Previous studies have shown that obese individuals experience a cascade of inflammatory responses. As an anti-inflammatory factor, the increased expression of IL-4 may be a compensatory mechanism aimed at maintaining cellular function, internal balance, and tissue integrity (42).

We observed no associations between ICAM-1, IL-8, IL-12p70, IP-10, MCP-1, MIP-1α, MIP-1β, E-selectin, P-selectin, and adolescent depression. Notably, ICAM-1, E-selectin, and P-selectin are adhesion molecules that are closely related to endothelial dysfunction by facilitating monocyte and lymphocyte attachment to endothelial cells. Endothelial dysfunction is a significant predictor of cardiovascular events associated with depression (43). Current research on adhesion molecules and depression primarily focuses on middle-aged and older adults, particularly those with comorbid cardiovascular diseases (44–47). This indicates that adhesion molecules may serve as age-related biomarkers of depression, necessitating further investigation into their roles in adolescent depression. IL-8, IP-10, MCP-1, MIP-1α, and MIP-1β are chemokines. Chemokines play significant roles in the nervous system, including regulation of neuroendocrine function, neurotransmitter systems, and neurodegeneration (48). Most studies on the relationship between chemokines and depression focus on adults, with few investigating adolescents. Byrne et al. reported no changes in peripheral blood IL-8 levels in adolescents with depression, which is consistent with our findings (18, 49). Additionally, a meta-analysis of adult depression revealed no significant changes in IL-8 levels (50). This lack of association may be attributed to sample size, medication status, and the heterogeneity of depression among participants. IL-12p70, the active form of IL-12, promotes T helper 1 responses and cell-mediated immunity while inhibiting T helper 2 cell differentiation. However, the evidence for IL-12p70 in adolescent depression is inconsistent. Pérez et al. reported increased peripheral blood IL-12p70 levels in adolescents with depression compared with healthy controls, whereas Byrne et al. reached a conclusion consistent with ours (18, 49). Further studies are needed to understand the relationship between IL-12p70 and adolescent depression.

We observed significantly elevated serum TNF-α, IL-4, and IFN-γ levels in adolescents with depression. Serum IL-4, IFN-γ levels may have diagnostic value in adolescent depression, but further validation with additional indicators is needed. Future research should focus on combining these markers with other serum inflammatory factors, particularly IL-6 and CRP.

Our study found a cross-sectional association between depression and inflammation in adolescent. However, previous research suggests a bidirectional relationship (51). On the one hand, early-life adversity (ELA) may lead to chronic inflammatory signaling in peripheral and central nervous systems by altering the immune system’s response to social stressors (52). Additionally, changes in the gut microbiota can influence depressive symptoms by modulating inflammation levels (53). The systemic inflammation induced by gut microbiota alterations can reach the central nervous system through various pathways, affecting inflammatory pathways (53). Peripheral inflammatory proteins entering the brain can increase the sensitivity of the cortico-amygdala circuit to threats, decrease the sensitivity of the cortico-striatal circuit to rewards, and alter executive control and emotional regulation in the prefrontal cortex, thereby triggering anxiety, depressive symptoms, and unhealthy behaviors (54). On the other hand, depressive symptoms can further exacerbate inflammation, creating a positive feedback loop (37). Longitudinal studies are necessary to investigate these associations further.

Except for cross-sectional study, Our study has other limitations. First, our sample size was insufficient, limiting the reliability of our conclusions. We intend to increase the sample size in future research to obtain more robust results. Secondly, we did not account for menstrual status in females, which could have affected inflammatory factor levels. Future studies should impose stricter restrictions on confounding factors such as menstrual history. Additionally, the levels of inflammatory factors in adolescents with depression may correlate with specific symptom clusters. Future research should use relevant symptom scales, such as atypical depression diagnostic scales, to evaluate atypical depressive symptoms in patients. Finally, data analysis was conducted from only a single center. Future studies should consider including multiple centers to enhance the generalizability of the findings.

## 5 Conclusion

In conclusion, adolescents suffering from depression exhibited notably higher serum cytokine levels compared to healthy controls, suggesting a greater immune activation, particularly in females. While cytokines show potential as diagnostic biomarkers for adolescent depression, further confirmation with additional parameters is required.

## Data Availability

The raw data supporting the conclusions of this article will be made available by the authors, without undue reservation.
